# Low prevalence of contraceptive counseling at Srinagarind hospital, Thailand among women of reproductive age with systemic lupus erythematosus

**DOI:** 10.1186/1742-4755-10-21

**Published:** 2013-04-11

**Authors:** Thannaporn Kittisiam, Yuthapong Werawatakul, Ratanavadee Nanagara, Orathai Wantha

**Affiliations:** 1 Department of Obstetrics and Gynecology, Faculty of Medicine, Srinagarind Hospital, Khonkaen University, Khonkaen, Thailand; 2 Division of Reproductive Health, Department of Obstetrics and Gynecology, Faculty of Medicine, Srinagarind Hospital, Khonkaen University, Khonkaen, Thailand; 3 Division of Allergy Immunology-Rheumatology, Department of Medicine, Faculty of Medicine, Srinagarind Hospital, Khonkaen University, Khonkaen, Thailand

## Abstract

**Objective:**

Unplanned pregnancy in women with SLE can have grave complications both for the child and the woman. We studied the prevalence of contraceptive counseling among women of reproductive age with SLE at a university hospital in Northeast Thailand.

**Methods:**

Recruited: 125 women with SLE, between 15 and 50 years, followed up at the Rheumatology Clinic. A questionnaire was administered and the results analyzed to identify the prevalence of contraceptive counseling.

**Results:**

The majority of women with SLE had had their reproductive goals evaluated (76.00%, 95% CI 66–83) and received contraceptive counseling (72%). Among the SLE patients at risk for pregnancy, only one-third used effective contraception and one-fifth of those did not have any background knowledge about SLE and pregnancy. Contraceptive counseling was more frequently given to women who had had a previous pregnancy or who were already concerned about SLE as related to pregnancy.

**Conclusion:**

The majority of SLE patients had at one time or other received contraceptive counseling, but some reported not grasping the gravity. The survey results presented herein suggest that a multidisciplinary team is needed to improve patient knowledge regarding SLE as it affects on pregnancy and relatedly contraceptive counseling.

## Introduction

Systemic lupus erythematosus (SLE) is a systemic autoimmune disease mostly found among women of reproductive age [1]. There is no official report on the prevalence of SLE among the Thai population. An epidemiologic study using a questionnaire survey of 2,463 subjects of rheumatic diseases in rural Thailand by Chaiamnauy et al. reported that the prevalence of CNTD was 8 in 10,000 [2]. In 2001, a community survey of musculoskeletal pain and management in Namphong District, Khon Kaen Province conducted by Auabandit et al. revealed the prevalence of SLE was 0.38% [3].

A 2009 study [4] of pregnancy outcomes among women with SLE at Srinagarind Hospital showed 37 women with SLE were pregnant in the 10 years between 1997 and 2006; 90% of whom had had SLE established before pregnancy. The disease was active during pregnancy in two-thirds and correlated with poorer pregnancy outcomes. In this group, the disease activity during pregnancy was a continuation from the pre-pregnancy period for 60% of the women; thus, ~40% of SLE patients became pregnant during an active disease phase.

During pregnancy, lupus improves in a third of women, remains unchanged in a third, and worsens in the remaining third. Thus, in any given pregnancy, the clinical condition can worsen or flare without warning. Common complications in a cohort of 13,555 women with SLE during pregnancy are pregestational diabetes, preeclampsia, renal failure, preterm labor, fetal-growth restriction, neonatal lupus, etc [5]. In general, pregnancy outcome is better if (1) Lupus activity has been quiescent for at least 6 months before conception (2) There is no active renal involvement manifest by proteinuria or renal dysfunction (3) Superimposed preeclampsia does not develop and (4) There is no evidence of antiphospholipid antibody activity [6]. For a better pregnancy outcome, women of reproductive age with SLE should receive preconception counseling and use contraception, hence they would know when is the right time that they can get pregnant. Due to the seriousness of the risks, we performed an observational study at Srinagarind University Hospital to determine (a) the prevalence of contraception counseling among women of reproductive age with SLE (b) the method of contraception used and (c) the factors associated with the use of contraception.

## Material and methods

Study population: Included in the study were 125 women between 15–50 year of age, either who were pregnant or not, in remission or still in the active phase, diagnosed SLE and being followed up at the Rheumatology Clinic, Srinagarind Hospital. The sample size was determined by a pilot study of 10 SLE women who achieved criteria. The volunteers were interviewed independently for about 30 minutes by an obstetrician or a rheumatology nurse. The questionnaire included questions on (a) SLE manifestations and activity (b) sociodemographics (c) obstetric and gynecologic history and (d) knowledge about reproductive health and basic knowledge about SLE disease as related to pregnancy.

All patients were diagnosed with SLE by a rheumatologist. These diagnoses were confirmed during a formal review of the medical records as per the Revised Criteria of the American Rheumatism Association for the Classification of Systemic Lupus Erythematosus (SLE).

The study procedures were approved by the Human ethics committee of faculty of medicine Khon Kaen University (June 24, 2011).

### Measures

Reproductive history was available for 125 participants. The information included (i) total number of pregnancies (ii) whether the pregnancy occurred after diagnosis of SLE (iii) whether the pregnancy was planned or not, (iv) the pregnancy outcomes (i.e., early miscarriage, induced abortion for any cause, late miscarriage or stillbirth) (v) any gynecologic operation (viz., tubal resection, hysterectomy or salpingectomy) and (vi) the menstrual pattern.

The volunteers were also interviewed about their educational status, marital status, income per month, disease duration and any disease activity (i.e., active with renal involvement, active without renal involvement or inactive disease). The medical records were reviewed for any indication of a potential teratogen used (i.e., MTX, MMF, cyclophosphamide or warfarin) or any evidence of thrombosis event (viz., stroke, myocardial ischemia, DVT, PE or renal vein thrombosis).

The 125 women with SLE were asked, “Have you ever been asked about your reproductive goals?” If yes, we determined the number of women who had been asked if they had received contraceptive counseling. We then evaluated the pregnancy risk. Women who were not sexually active (have not had sexual intercourse for at least 3 months), were in menopause or had had a hysterectomy or tubal resection were not at risk for pregnancy; all others were defined as at risk for pregnancy. This group were not queried about their knowledge or attitudes because they did not need contraceptive counseling anymore.

All women were asked about the method of contraception used. We then evaluated the effectiveness of the method: hormonal contraception and IUD method counted as effective contraception, even if not used consistently. Women who used ineffective contraception were evaluated for their background knowledge of SLE disease as it relates to pregnancy via 3 questions: (1) “Did you know that women with SLE can get pregnant and that the medications that control the disease or the disease itself may affect the pregnancy outcome?” (2) “Did you know that pregnancy can aggravate SLE activity?” and (3) “Did you know that women with SLE should avoid getting pregnant if the disease has been inactive for less than 6 months?” Woman who could not answer at least 2 of the 3 foregoing questions were considered as ‘not having background knowledge of SLE disease as related to pregnancy’.

In women who did not use contraception, the reasons for not using it were recorded. Among women who had been asked about their reproductive health, we evaluated their perception of contraceptive counseling with the following questions: (1) “Have you ever received any contraceptive counseling?” (2) If so, “Did you understand the counseling given?” The women not at risk for pregnancy were categorized as having correctly understood the risks because they did not need contraceptive counseling anymore.

The pregnant women enrolled in this research were asked (1) whether they had received contraceptive counseling (2) the method of contraception they used before pregnancy (3) their background knowledge regarding SLE disease as related to pregnancy and (4) their intention for pregnancy.

### Statistical analysis

The sociodemographic characteristics, SLE manifestation and activity, obstetric- gynecologic history, history of getting contraceptive counseling and the knowledge of SLE disease related to pregnancy of the study volunteers are reported using frequencies, percentages, means and standard deviations as appropriate.

The women at risk for unplanned pregnancy group were further analyzed using univariate logistic regression to identify the factors associated with effective contraception use and associated with receiving contraception counseling. Variables included age, income, educational status, taking teratogenic medication, active disease with renal involvement, disease duration, healthcare provider (i.e., rheumatologist or obstetrician), history of prior pregnancy and bad obstetric outcome (e.g., induced abortion, other pregnancy complication) and patient, partner and the family’s desire to have a child. With each associated variable, the multivariate logistic regression was run anew.

## Results

A total of 125 women of reproductive age (15–50 years) with SLE were interviewed. The sociodemographic characteristics, SLE disease activity and disease’s related complications are summarized in Table 1. Over one-half of the lupus patients were married or living with a partner (56.8%). One-third (33.6%) had active disease with renal involvement, whereas 8.0% and 21.6%, respectively, had a history of thrombosis and potential teratogenic drugs use.

**Table 1:** Socio-demographic characteristics and reproductive histories of women between 15 and 50 years with SLE

**N = 125**	**Frequency/mean ± SD**	**Percent/range**
Age, years	33.48 ± 9.3	15-50
Disease duration, years	8.4 ± 6.9	1-35
Education		
• Less than elementary education	27	21.6%
• Secondary education	13	10.4%
• Less than or equal to high school	40	32.0%
• Bachelor degree	38	30.4%
• Master degree/Doctorate	7	5.6%
Marital status		
• Married/living with male partner	71	56.8%
• Divorced	6	4.8%
• Widowed	2	1.6%
• Not married	46	36.8%
Income		
• < 10,000 THB per month	85	68.0%
• 10,000-30,000 THB per month	35	28.0%
• > 30,000 THB per month	5	4.0%
SLE activity in the past 6 months		
• Active with renal involvement	42	33.6%
• Active with minor organs involvement	17	13.6%
• Inactive	66	52.8%
History of thrombosis		
• Yes	10	8.0%
• No	115	92.0%
Potential teratogenic drug use		
• Yes	27	21.6%
• No	98	78.4%
Hysterectomy	3	4.23%
Ever pregnant	71	56.8%
Total pregnancies among those ever pregnant	1.97 ± 0.92	1-5
Being pregnant, unplanned	7	5.60%
Women pregnant after diagnosed SLE	24	19.24%
• Total pregnancies after diagnosed SLE	1.46 ± 0.78	1-4
• Unplanned (n = 13)	1.31 ± 0.85	1-4
Live births (n = 56)	1.91 ± 0.82	1-5
Early miscarriages (n = 12)	1.083 ± 0.29	1- 2
Induced abortions (n = 11)	1.09 ± 0.30	1-2
• Uncontrolled severe SLE	6/11	54.55%
• Socioeconomic/psychosocial cause	5/11	45.55%

The reproductive history of each patient is also summarized in Table 1. More than one-half of the patients had been pregnant (71; 56.8%): of the 24 (19.24%) who got pregnant after diagnosis of SLE; half of them (10.4%) were unplanned pregnancies. Therapeutic abortion was indicated for 11 (8.8%) patients; 6 cases due to uncontrolled active SLE (3 unplanned vs. 3 planned pregnancy) and 5 due to socioeconomic or psychological reasons.

**Table 2:** Pregnancy risk of woman age < 50 years with SLE

**Pregnancy risk and intention (n = 125)**	
• Not at risk; three variables overlapped	82/125 (65.6)
○ Menopause	26/125 (20.8)
○ Not sexually active in last 3 months	50/125 (40.0)
○ Other medical/surgical conditions (included TR, hysterectomy)	25/125 (20.0)
• At risk for pregnancy	43/125 (34.4)
○ Pregnant	7/125 (5.6)
○ Trying to become pregnant	1/125 (0.8)
○ Not trying to become pregnant OR not considered	35/125 (28)

SLE patients were categorized by risk of pregnancy (Table 2). A majority (82/125; 65.6%) of the SLE patients followed up had no risk for pregnancy, either because of their postmenopausal status, not being sexually active for at least 3 months, or the existence of some other co-morbid condition obviating pregnancy. Some patients may have had more than one factor. A minority of patients (43/125; 34.4%) were at risk for pregnancy; among whom most (35/43; 81%) were not worried about whether or not they got pregnant so they were defined as women at risk for unplanned pregnancy.

Contraception use among women at risk for unplanned pregnancy is shown in Table 3. Only 37.1% reported use of effective contraception (viz., hormonal method or IUD). However, less than one-half of them reported consistent use of contraception. Another worrisome factor was the misunderstanding of the women at risk for unplanned pregnancy that they could not get pregnant because of their having a chronic disease, or infrequent sexual intercourse so there was no need for a contraceptive.

**Table 3:** Contraception use among women at risk for unplanned pregnancy (n = 35)

**Frequency of contraceptive use in past 3 months**	
• Never	12/35 (34.29%)
• Sometimes	7/35 (20.00%)
• Always	16/35 (45.71%)
Method of contraception used in past 3 months	
• None (including withdrawal and rhythm)	12/35 (34.29%)
• Combined oral contraceptive pill	7/35 (20.0%)
• Progestin injection	3/35 (8.57%)
• Progestin implantation	2/35 (5.71%)
• IUD	1/35 (2.86%)
• Condom	10/35 (28.57%)
Reasons for not using contraception among those not trying to get pregnant (n = 12)	
• Believe that they could not get pregnant because of having a chronic disease	3/12 (25.0%)
• Believe that infrequency of sexual intercourse obviated the possibility of getting pregnant	2/12 (16.66%)
• Others	7/12 (58.33%)

Contraception use and counseling among women of reproductive age with SLE is presented in Table 4. Some 24.0% (30 of 125) of the participants had never been asked about their reproductive goals or had forgotten, 5.6% (7 of 125) of these were at risk for pregnancy. A small 3.2% (4 of 125) of this group did not use contraception and 1.6% (2 of 125) did not have basic knowledge about SLE disease as related to pregnancy. Of the 125 women with SLE, 95 (76.8%; 95% CI 68–83) reported having been asked about their reproductive goals: 36 (28.8%) were at risk for pregnancy (1 of them tried to become pregnant); 1 (0.8%) reported she did not know about or use effective contraception to prevent aggravation of the SLE. Some 3.2% (4 of 125) had been asked about their reproductive goals, whether they were at risk for pregnancy, whether or not they used contraception and their knowledge about SLE as related to pregnancy.

**Table 4:** Contraceptive use, counseling and basic knowledge of SLE as related to pregnancy among women with SLE of reproductive age (n = 125)

	**Frequency**	**Percent**
Had been asked about desire to be pregnant	95	76.8 (95%CI 68–83)
Not at risk for pregnancy	59	47.2
At risk for pregnancy (either planned or unplanned)	36	28.8
Using effective contraception	13	10.4
Using ineffective contraception**	11	8.8
Have knowledge*	10	8.0
Do not have knowledge	1	0.8
Non-contraceptive use	12	9.6
Have knowledge*	8	6.4
Do not have knowledge	4	3.2
Had never been asked about desire to get pregnant	30	24.0
Not at risk for pregnancy	23	18.4
At risk for pregnancy	7	1.56
Using effective contraception	3	2.4
Using ineffective contraception	0	0
No contraception	4	3.2
Have knowledge	2	1.6
Do not have knowledge	2	1.6

* Effective contraception means using any hormonal method or IUD, unless it was already defined as ineffective; ** Ineffective contraception means using a condom. Other methods including rhythm and withdrawal. were categorized as non-contraception.

Most (90/125; 72.0%; 95% CI 63–79) remembered having been given contraceptive counseling, and only 39 (11.2%; 95% CI 6–18) reported not having understood. Since they did not need any contraceptive counseling, we included all the women in the ‘received contraceptive counseling group’ who were: in menopause, had had tubal resection or hysterectomy, or were not sexually active. The majority of counseling providers were rheumatologists (50.4% vs. 8.0%) (Table 5).

**Table 5:** Perception of contraceptive counseling among woman between 15 and 50 years with SLE

**N = 125**	**Frequency**	**Percent (95% CI)**
Had ever been asked about their reproductive health/goals?		
• Never	29	23.2
• Yes	96	76.8 (68–83)
Among those asked, Had they ever received contraceptive counseling?		
• Never	4	3.2
• Forgot	2	1.6
• Yes*	90	72.0 (63–79)
Level of understanding among women who had received contraceptive counseling (n = 90)		
• Understood	34	60.8 (52–69)
• Not well	39	11.2 (6–18)
Counseling provider among women who had received contraceptive counseling (n = 90)		
• Rheumatologist	63	50.4 (41–59)
• Obstetrician	10	8.0 (3–12)

* Included the women who were in menopause, not sexually active, had undergone TR or hysterectomy.

In Table 6, we focused on pregnant women. One woman (0.8%) reported that she had never been asked about her reproductive health or goals, did not use contraception, did not have any background knowledge about SLE disease as related to pregnancy and had an unplanned pregnancy. Six of the 125 (4.8%) women reported they had been asked about their reproductive goals, 4 (3.2%) used contraception vs. 2 (1.6%) who did not.

**Table 6:** Contraceptive counseling among being pregnant women with SLE 15–50 yearsn = 125

	**Frequency**	**Percent**
Being pregnant	7	5.6
Had never been asked about desire to get pregnant	1	0.8
No contraception	1	0.8
Had been asked about desire to get pregnant	6	4.8
Using effective contraception but inconsistent	3	2.4
Using ineffective contraception	1	0.8
No contraception	2	1.6

Among the pregnant women who had been asked about and used contraception, 1 (0.8%) used ineffective contraception (condom), did not have knowledge about SLE as related to pregnancy and had a planned pregnancy whereas 3 (2.4%) used effective contraception (COC) albeit inconsistently, had knowledge but had an unplanned pregnancy. We found that among the women who had been asked, 2 (1.6%) did not use contraception, did not have knowledge, and had an unplanned pregnancy.

We categorized 35 women as ‘at risk for unplanned pregnancy’ if (a) they did not try to get pregnant or (b) did not consider getting pregnant (Table 2). We then performed univariate logistic regression to evaluate the variables associated with using effective contraception and receiving contraceptive counseling (Table 7). Four of the 17 variables were associated with receiving contraceptive counseling—including prior pregnancy and 3 variables on basic knowledge of SLE disease as related to pregnancy. But when further analyzed using multivariable logistic regression, the 3 knowledge variables were not statistically significant (Table 8).

**Table 7:** Univariable predictors of effective contraception use and receiving contraceptive counseling among women with systemic lupus erythematosus at risk for unplanned pregnancy*

	**n**	Use of effective contraception***	**Receive contraception counseling**
Age > 30 (vs. ≤ 30)	15	1.24 (0.31-4.93)	0.69 (0.14- 3.35)
Income ≥ 10,000 THB (vs. <10,000)	8	2 (0.4-9.9)	2.45 (0.25-23.6)
College degree (vs. less education)	10	1.18 (0.26-5.34)	1.26 (0.21-7.65)
Taking teratogenic medication	8	0.17 (0.02-1.65)	0.85 (0.14-5.39)
Active renal disease (vs. stable disease)	11	0.24 (0.04-1.46)	0.32 (0.05-1.87)
Active non-renal disease (vs. stable disease)	5	0.74 (0.09-5.49)	0.75 (0.06-9.26)
Disease duration > 5 yr (vs. < 5 yr)	13	1.09(0.25-4.50)	2.06(0.35-12.16)
Know about the effect of SLE on pregnancy	27	0.98 (0.19-5.00)	13.33 (2.05-86.34) ¥
Know about the effect of pregnancy on SLE	29	0.52(0.09-3.10)	12.50 (1.69-92.25) ¥
Know about the better prognosis if pregnancy occurs after 6 months of being stable or free of disease	21	1.10 (0.27-4.50)	20 (2.08-192.64) ¥
healthcare provider			
• Rheumatologist (vs. no provider)	23	0.88 (1.16-4.71)	-
• OB	4	1.66 (0.14-18.87)	
Prior pregnancy	24	3.8 (0.67-21.47)	13.2 (2.03-85.81) ¥
Prior induced abortion	5	3 (0.43-20.9)	-
Not desire to get pregnant (vs. desire or not considered)			
• Women with SLE	25	0.47 (0.08-2.75)	3.5 (0.47-24.65)
• Their partners		1.18 (0.19-6.66)	1.26 (0.17-15.31)
• Their families	10	0.50 (0.07-3.42)	2.64 (0.29-19.51)
	27		

* Values are the odds ratio (95% confidence interval) unless otherwise indicated. ** Age and disease duration reported using mean difference & 95% confidence interval. ***Effective contraception included hormonal methods and IUD. ¥ = significant (P < 0.05).

**Table 8:** Multivariate predictors for receiving contraceptive counseling among women with systemic lupus erythematosus at risk for unplanned pregnancy*

	**Odds ratio**	**95%CI**
Know about the effect of SLE on pregnancy	2.80	0.26- 29.47
Know about the effect of pregnancy on SLE	3.41	0.28- 41.39
Know about the better prognosis if pregnancy occurs after 6 months of being stable or free of disease	10.34	0.91-118.05

## Discussion

The definition of contraceptive counseling is using of interpersonal communication skills to effectively provide information that will help the woman decide on her contraceptive needs. From a rheumatological perspective, the first step is to ask the woman about her reproductive desires or goals: does she or do she and her partner/family want to get pregnant. In our research, 76% of the women reported that they had been asked this sort of question and 28.8% of them were at risk for pregnancy although only one-third of them used effective contraception. Another 18.4% of the women either used no contraception or ineffective contraception and one-fifth of this group did not have basic knowledge about SLE as related to pregnancy. We did not ask when the last time that they had received contraceptive counseling was so we cannot define whether the counseling affected contraceptive use or not.

In the questionare, we questions about “not sexually active”, it was uncertained that they still not sexually active in the future. In this research, we defined them as “not at risk for pregnancy” at the time we studied. However, this group should also received contraceptive counseling to prevent unplanned pregnancy.

Although most women at risk for unplanned pregnancy use contraception, barrier methods with a high rates of failure [7] were the most commonly used, even among those with a history of thrombosis. Importantly, we found that in the non-contraception group at risk for unplanned pregnancy, some did not even realize that they could become pregnant and thus thought that contraception was unnecessary. Moreover, most of being pregnant women had ever received contraceptive counseling, but got unplanned pregnancy. Clearly, patients need more background knowledge about their reproductive health as it relates to SLE and how to appropriately use contraception.

In the current study, we found that only a small number of women were referred to the Family Planning Unit. Four variables were associated with receiving contraceptive counseling (a) know that women with SLE can get pregnant (b) know that the medications controlling the disease can affect the pregnancy outcome (c) know that pregnancy itself can aggravate SLE and (d) have been pregnant before, but when we analyzed the first three dependent variables in a multivariate logistic regression, they were not statistically significant—perhaps because they confounded each other. Effective counseling nevertheless needs to convey the basics of SLE disease as related to pregnancy.

The benefit of study is to make physicians and nurses aware of the need for contraceptive counseling for women with SLE, including other medical diseases. The limitation for a study with a secondary objective was the small sample size; consequently, the outcome of associated factors could not be fully analyzed. Further study may be required. For Women using hormonal contraception inconsistently should not be considered as using “effective” contraception, it might reduce the generalizability of the paper.

According to a previous study at the University of California [8], the only significant predictive factor for receiving contraception counseling was a prior pregnancy. The other important variable was healthcare provided by an obstetrician-gynecologist; possibly because women prefer getting contraceptive counseling from an obstetrician rather than a rheumatologist. In the current study, we could not make any conclusions about this variable because of the different proportion of patients served between obstetrician and rheumatologist. Nevertheless, both the attending rheumatologist and the consulting obstetrician should encourage women with SLE at risk for unplanned pregnancy about the interactions of SLE disease as related to pregnancy. At Srinagarind Hospital, 90% of pregnant women with SLE knew their diagnosis before getting pregnant. Two-thirds had active lupus during their pregnancy and 40% had active lupus even before conception and the pregnancy outcomes were grave [4]. According to a 2010 report in Autoimmunity Reviews, [9] lupus pregnancies can be successful, so physicians should introduce a discussion with the patient about pregnancy and its problems and a multidisciplinary team (including rheumatologists, internists, obstetricians and neonatologists) should take care of the patient. In the current study, a very small proportion of patients were referred to family planning, so cooperation of the rheumatologist and obstetrician might reduce the number of unplanned pregnancies. Moreover, the counseling should probably not be just once but annually or even more frequently in the active renal disease group.

The March 2010 IPPF Medical Bulletin [10]. New Recommendations for Contraceptives for Women with SLE indicated that SLE is one of the conditions that makes unintended pregnancy an unacceptable health risk and will happen in women at their peak reproductive age. The role of providers is to assist these women with providing appropriate information to make decisions on pregnancy and when they do make a decision to have a baby, ensure that this is timed when there is less risk and better follow up.

The important policy is all providers not only the Ob-Gyn should have an update on contraception, counseling skills and perhaps develop a protocol that regardless of who sees these patients at first visit should start assessing risk for pregnancy and help the patient get to the right person to get effective counseling.

However, the appropriate contraception methods, consistency use and complication of contraception use were not reviewed, so the further study required.

## Competing interests

The authors declare that they have no competing interests.

## Authors’ contributions

All authors read and approved the final version to be submitted for publication. TK, MD had full access to all of the data in the study and takes responsibility for the integrity of the data and the accuracy of the data analysis. Study conception and design. TK, YW, RN. Acquisition of data. TK, OW. Analysis and interpretation of data. TK, YW, RN.
